# Frictional Properties of Biomimetic Micro-Hexagonal-Textured Surfaces Interacting with Soft Counterfaces under Dry and Wet Conditions

**DOI:** 10.3390/biomimetics9090542

**Published:** 2024-09-07

**Authors:** Zain Eldin Qatmeera, Agnes Bajjaly, Haytam Kasem

**Affiliations:** Department of Mechanical Engineering, Azrieli College of Engineering, Jerusalem 9103501, Israel; zainkatmera@gmail.com (Z.E.Q.); bajjaly.agnes@gmail.com (A.B.)

**Keywords:** friction, biomimetic, micro-hexagon, textured surface, soft counterface, gelatin, chicken skin

## Abstract

Biomimetic micro-hexagonal-textured surfaces have sparked interest for their application in fields that demand high friction and adhesion, such as micro-robotics and biomedicine. Despite extensive research conducted on this specific microstructure, its friction behavior against soft counterfaces remains a topic that has not been fully investigated yet. This study examines how micro-hexagon textures behave when they come into contact with engineered and biological materials like gelatin and chicken skin in dry and wet conditions. The results show clearly that under dry contact conditions, flat surfaces generate higher friction compared to hexagon micropattern surfaces. Under wet conditions, hexagon micropattern surfaces generate higher friction compared to flat surfaces. In wet conditions specifically, the static coefficient of friction is up to 13 times greater than that of a flat specimen against glass, up to 11 times greater against gelatin, and up to 6 times greater against chicken skin. For the dynamic coefficient of friction, the patterned surface demonstrates a maximum increase by a factor of 28 against glass, 11 against gelatin, and 5 against chicken skin. These results further develop our knowledge of these hexagon micropattern surfaces and pave the way for their utilization in future technological advancements in which soft and wet counterfaces are to be considered, such as in biomedical applications that can benefit from increased friction in wet conditions for better control and stability.

## 1. Introduction

Throughout millions of years of evolution, every species has adapted to its surroundings and evolved to devise incredibly effective solutions to the obstacles they encounter on a daily basis, often surpassing human creativity. Engineers and scientists have been interested in studying these evolutionary adaptations devised by nature [1], as they can apply and mimic these principles to solve various complex human engineering problems. This practice, known as biomimicry, is the study and imitation of natural biological designs, processes, and systems. It has yielded remarkable and pivotal advancements in various engineering fields, such as robotics [2], architecture [3,4], medicine [5], materials [6], and other engineering fields [7,8]. One field in which biomimicry has achieved important advancements is tribology [9,10], which is the study of friction, wear, and lubrication. Temporary attachment systems of various animals like geckos [11,12], beetles [13,14,15], and tree frogs [16,17,18,19] have been examined and mimicked for their amazing adhesive and frictional characteristics, even on uneven surfaces.

Temporary biomimetic attachment mechanisms inspired from nature can be divided into two main categories that have been studied by scientists for their frictional and adhesive properties [20]. The first category is hairy attachment systems, such as spatula setae contact elements inspired by geckos, suitable for shear-induced short-term adhesion [21,22,23], and mushroom-shaped contact elements inspired by the sticky hairs found in male beetles from the Chrysomelidae family, suitable for passive long-term adhesion [24,25,26,27,28], In addition, combined micro-mushroom and spatula setae structures were also examined [29]. The second category comprises textured pads suitable for attachment under wet conditions, such as the micro-hexagons inspired by the contact elements of tree and torrent frogs [16,17,18,19]. This second category is the focus of the present study, in which the micro-hexagon attachment system will be further explored, particularly when interacting with soft counterface materials, whether biological or engineered.

As a result of natural evolution and adaptation, the contact area on the tree frog attachment pads is divided into many micro-hexagons separated by narrow micro-channels [30]. The hexagonally sub-divided pads are largely composed of keratin, a fibrous protein that provides strength and flexibility, while the outer layers of the pads comprise specialized epidermal cells [31]. These hexagonally patterned cells are adapted to create a large contact area with antagonist surfaces, in which each hexagonal element has relatively independent movement of the others, to fit and conform to the roughness of the mating surface. This adjustment enhances the adhesion force by maximizing the effective contact area. Moreover, the micro-channels separating these micro-hexagonal elements play a crucial role in the overall structure. They serve as channels for liquids, allowing fluid to move out of the contact area when adhering to a wet surface due to the generated squeeze, this increases the friction between the patterned surface and the antagonist counterface, demonstrating the effectiveness of the micro-hexagon pattern to generate friction under wet conditions and preventing hydroplaning [18,32].

It is well established that the biological tree frog attachment system mainly functions by wet adhesion, due to the presence of a thin fluid film between the mating surfaces. The generated adhesive forces are believed to originate from both capillary and viscous hydrodynamic dependent interactions [33,34,35,36]. However, according to Langowski et al. [16], the contribution of van der Waals forces cannot be excluded. In addition, hexagonal microstructures are well known for their ability to generate friction forces, particularly under wet conditions [37,38]. One of the key parameters at the origin of this unique frictional performance of tree frog hexagonal microstructures is their extremely soft structures, which enhances the close contact with the opposite substrate [31].

Multiple studies have investigated biomimetic hexagonal-patterned surfaces. For example, Varenberg et al. examined the effect of varying geometric parameters of hexagonal micropatterns on the friction force when interacting with a rigid glass counterface [32]. In another study, Drotlef et al. investigated the influence of wetting properties on adhesion and friction forces of hexagonally micropatterned surfaces against a spherical glass counterface [39]. Additionally, Meng et al. explored tree frog adhesion biomimetics, providing insights into the potential for new smart adhesives that perform effectively under wet conditions [18]. To the best of our knowledge, all studies evaluating this pattern against counterfaces have considered smooth and rigid flat materials, such as glass or epoxy, except for one study by Tsipenyuk et al. in which the authors focused on the effect of varying the geometry of micro-hexagonal-structured surfaces on the frictional coefficient when tested against human hand skin in the presence of shaving foam [40].

In nature, tree frog attachment pads maintain their exceptional tribological performances (friction and adhesion) on a variety of rough and soft surfaces, enhancing their ability to hunt, escape predators, and move efficiently in various environments. In this spirit, the present research delves deeper into the interactions of micro-hexagonal-patterned surfaces with soft counterfaces, such as gelatin and chicken skin. This study examines the friction forces generated in both wet and dry contact conditions, comparing these interactions to those of a flat reference surface made from the same materials tested under identical contact and operational conditions. The results demonstrate the potential of these micro-hexagonal structures to be utilized in a wide variety of future applications, particularly in fields such as micro-robotics and biomedicine, in which stable and relatively high friction is required when contacting soft, rough, and wet counterfaces, thus representing a pivotal advancement.

## 2. Materials and Methods

In this study, both the static and dynamic friction coefficients of a micro-hexagonal-structured surface and a flat reference made from the same soft elastomeric PVS material were examined against multiple soft counterfaces, such as gelatin and chicken skin. The results were also compared to the equivalent friction coefficients obtained against a rigid glass counterface when tested under both wet and dry conditions.

### 2.1. Micropatterned and Smooth Reference Samples

The micro-hexagonal-structured specimen was prepared by pouring two components of Polyvinyl Siloxane (PVS) onto a negative template wafer imprinted with a micro-hexagonal pattern. The hexagonal-patterned templates have a diagonal length of 50 µm, a spacing of 10 µm (microchannel width), and a microchannel depth of 10 µm. These templates were made from Si wafers using the photolithography process [41], which can produce high-quality, well-defined, and accurate micropatterned negative molds, as described in references [39,40]. This precision is essential for producing accurately patterned specimens necessary for conducting reliable tribological tests.

**Figure 1:**
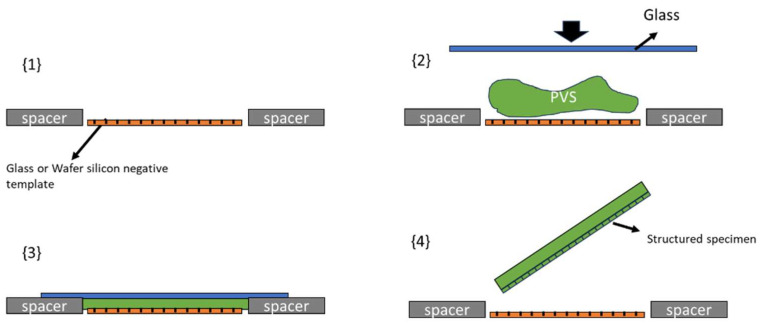
Schematic description of PVS hexagonal-micropatterned specimen preparation.

**Figure 2:**
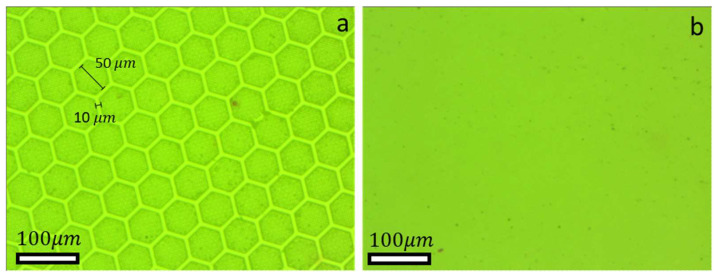
Optical microscope images: (**a**) patterned specimen with micro-hexagonal structure; (**b**) flat reference specimens.

The preparation process of the patterned samples, as described in reference [25], is illustrated schematically in Figure 1. First, two glass spacers were placed next to the negative template wafer to ensure uniformity in the thickness of the final specimen produced (1 mm) {1}. Next, PVS was poured over the negative template wafer in its liquid phase {2}, then another glass plate was placed on the top of the spacers to unify the sample thickness. The system was kept in this state until the PVS completely solidified {3}. Once solidification is complete, the glass is removed, and the ready-to-use micro-hexagonal-patterned specimen (see Figure 2a) can be released {4}.

The flat reference specimen (Figure 2b) was prepared in a manner similar to that of the patterned specimen. However, instead of pouring the PVS onto a negative wafer template, it was poured onto a glass surface with an average roughness R_a_ of 30 nm. This process yielded a remarkably flat and smooth, PVS surface.

### 2.2. Counterface (Substrate)

Tests were conducted to examine the friction coefficient of the micro-hexagonal-structured surface against various soft and hard counterfaces. Two soft counterfaces were used in this study: (i) gelatin that was extracted from sausage casings and that has an elastic module ranging between 10 and 100 KPa, as mentioned in reference [42], and (ii) biological chicken skin, which was extracted from fresh chicken obtained locally and that has an elastic module of approximately 30 Kpa, as stated in reference [43]. Gelatin was chosen as the first soft counterface, because it is a man-made material with consistent and controllable properties that can mimic the mechanical properties of biological tissues. Chicken skin was chosen as the second soft counterface due to its availability, ease of handling, and proven effectiveness in mimicking biological tissue as described in references [44,45].

**Figure 3:**
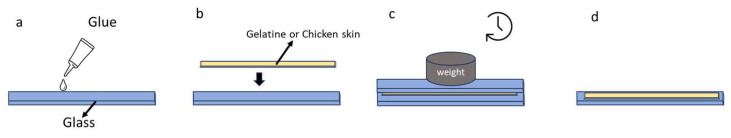
Schematic description of counterface preparation: (**a**) application of glue; (**b**) placement of gelatin stipe; (**c**) gelatin flattening using a second glass plate and weights; (**d**) ready to use gelatin counterface.

The process of preparing the gelatin strip involved soaking it in water for one minute. Afterwards, it was lightly patted with tissue to remove excess moisture. A small amount of glue was applied to a glass plate (Figure 3a), and then the gelatin strip was placed on top of the glue (Figure 3b). Another glass plate was positioned over the strip, with weights placed on top to ensure flattening and adhesion (Figure 3c). Once the glue dried completely, the glass plate and weights were removed, leaving the gelatin strip securely attached to the glass plate (Figure 3d).

The chicken skin was prepared in a similar manner but without presoaking it in water. First, the chicken skin strip was lightly patted with tissue to remove moisture then a small amount of glue was applied to a glass plate; afterwards the chicken skin strip was placed on top of the glue. Another glass plate was placed over the chicken skin strip, along with weights. After the glue dried, the chicken skin remained securely attached to the glass plate, ready for testing (Figure 3d).

In addition, a hard counterface made of a flat and rigid glass plates was used as well, for the purpose of making a comparison. The glass plate used had dimensions of 76 mm × 26 mm × 1 mm and was purchased from Am Woellerspfad 4, Paul Marienfeld GmbH & Co. KG, Lauda-Königshofen, Germany. The glass plate had a smooth surface with an average roughness R_a_ of approximately 30 nm (see Table 1), as measured using a 3D optical profilometer (NT1100, Wyko, Greenback, TN, USA).

### 2.3. Experimental Set-Up

**Figure 4:**
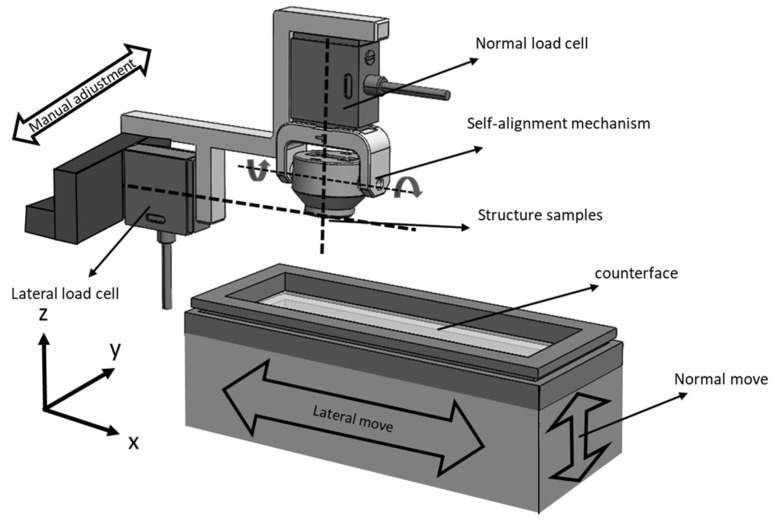
Schematic illustration of the used customized bi-axial tribometer.

In this study, friction tests were conducted using a customized biaxial tribometer specially designed in the Tribology and Microstructure Laboratory at Azrieli College of Engineering in Jerusalem. The device comprises two main operational components: (i) The lower drive unit encompasses three translation stages, two motorized and one manual, facilitating movement in all three directions of the stage holding the counterface as shown in Figure 3. Movements in the normal (z) and lateral (x) directions are achieved using (ZABER motors X-LSM025A-E03, Vancouver, British Columbia, Canada) (25 mm total travel distance and 15 µm accuracy) and X-LHM050A-E03 (50 mm total travel distance and 75 µm accuracy) for normal and lateral displacement, respectively. While adjusting the contact location between the mating surfaces, the third (y) direction is obtained manually using an accurate micrometric translation stage (ZABER model TSB28M-MH2). (ii) The stationary measurement unit located at the top of the device, which includes two-high resolution (0.1 mN) load cells (FUTEK’s FSH00092-LSB200, Irvine, CA, USA) that provide accurate measurement of force variation in both the vertical and lateral directions in the interface during a friction experiment. These sensors are positioned perpendicularly to each other, ensuring that their lines of action intersect precisely at the interface location, thereby preventing any undesired torque (see Figure 4). The measurements were conducted using a multifunctional data acquisition board (Lab-PC-NI USB-6211, National Instruments Corporation, Austin, TX, USA), and the data were processed using the LabVIEW 2017 software platform (11500 N Mopac Expressway, National Instruments Corporation, Austin, TX, USA). The data acquisition sampling rate was set to 1000 samples per second, and subsequent averaging was performed every 100 points to reduce noise.

Ensuring precise alignment of mating surfaces during testing is critical for an accurate tribological analysis. To achieve this, a passive self-alignment system was used based on the principle of two free-rotation axes. This system facilitates full flat-on-flat contact between mating surfaces, maximizing parallelism between the mating surfaces. It comprises a circular frame manufactured using 3D printing technology (PlasCLEAR material, Asiga, Alexandria, Australia) and incorporates two small metallic axes to allow freedom in the two axes perpendicular to the vertical axis. Further explanations of the used tribometer and its operation can be found in references [25,46]. The experimental set-up allowed for the customization of various operational parameters, including counterface sliding velocities, applied normal force, sliding distance, and dwell time as well, through a user-friendly programmed interface.

### 2.4. Operational Conditions

The samples tested, patterned and flat reference, are both made of PVS material, an elastomer known for its great flexibility with an elastic modulus around 3 MPa [32]. Using such a soft elastomer helps create highly compliant structures that enhance adhesive and frictional performance. These compliant structures store minimal elastic repulsive energy, allowing them to conform closely to the roughness of the counterface, thereby increasing the intimate contact area and the resulting adhesive and friction forces. Given this flexibility, along with the presence of water in the contact, it is hence judicious to examine the effect of the sliding velocity on the frictional behavior of the patterned surfaces. Therefore, tests were conducted at sliding velocities of 5, 7.5, and 10 mm/s. All tests were conducted under a vertical load of F = 5 N, leading to a nominal contact pressure of P = 130 KPa. For each speed and respective surface and counterface combination, the test was repeated 13 times under dry conditions and 13 times under wet conditions. In each category, the results of the first 3 repetitions were considered as tests and were discarded to ensure data consistency, while only the last ten repetitions were saved for analysis. Regarding the number of repetitions conducted in this experiment, it is widely accepted to conduct a limited number of repetitions (between 5 and 10) for such investigations, provided good repeatability is obtained.

### 2.5. Experiment Procedure

**Figure 5:**
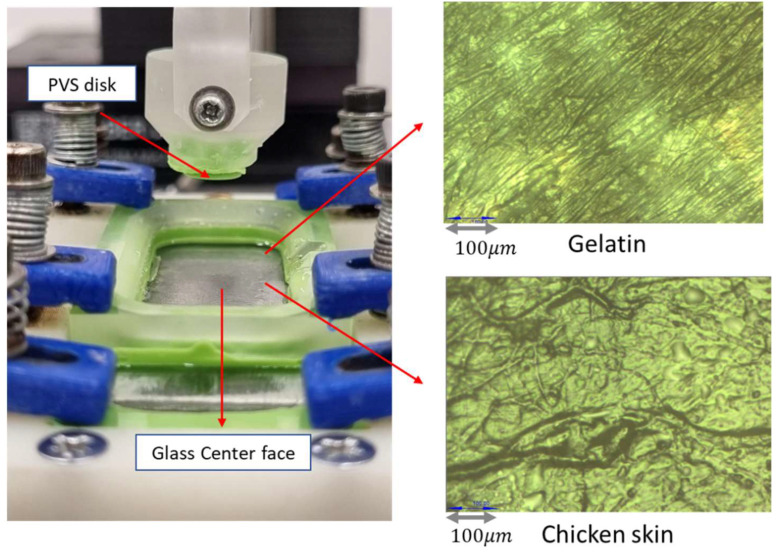
Close view of the friction pair and optical image of the tested counterfaces, i.e., gelatin or chicken skin.

The experimental procedure involves several sequential steps. First, PVS specimens (whether patterned or smooth reference) were prepared to meet specific requirements for the experiment, ensuring surface cleanliness to eliminate any impurities that may affect the results. Next, the ready-to-use PVS specimens were cut into small disks with a diameter of 7 mm and height of 1 mm and then attached to the sample holder. Additionally, the previously prepared glass plates glued with the respective counterfaces (gelatin and chicken skin strip), along with a plain glass plate for comparison, were mounted on the lower moving stage of the tribometer, as depicted in Figure 5. Subsequently, the prepared counterface was surrounded with 3D-printed protective borders around the contour of each counterface to create a dedicated soaking area for the tested liquid to meet the needs of the specific tests. Once the samples, hexagonal-patterned or flat reference, were mounted on the up holder and connected to the self-alignment system, the friction test could be initialized under dry or wet conditions according to the chosen configuration and selected operational conditions. Finally, the software could be initiated to commence the experiment, managing the entire process (repetition of 13 friction cycles).

**Figure 6:**
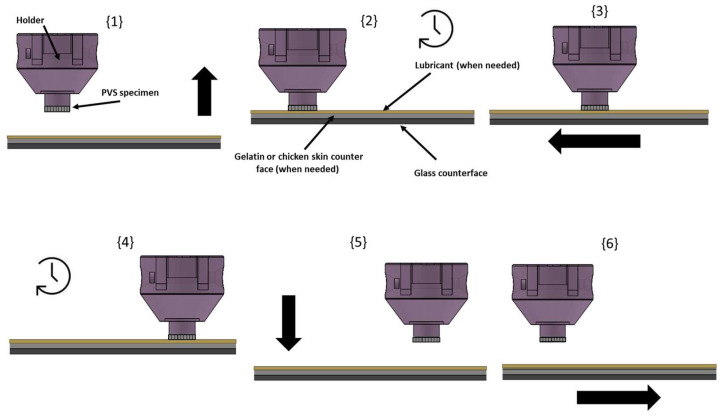
Schematic illustration of the different successive stages followed during a single friction test cycle.

The experiment took place following six successive stages, as described in Figure 6 below:

- Stage {1}: The counterface is brought gradually into contact with the PVS textured specimen at a speed of 1 mm/s, increasing the load until a 5 N load is reached and maintained between the mating surfaces for the entire test.
- Stage {2}: Upon reaching the 5 N vertical load, horizontal sliding motion initiates immediately.
- Stage {3}: Horizontal movement of the counterface is executed at a constant sliding speed. Experiments were conducted with the same combination of surface and counterface, but at three different constant speeds of 5, 7.5, and 10 mm/s. The travel distance during this stage is set to 20 mm, and the friction force resisting sample motion is recorded and saved.
- Stage {4}: Once horizontal movement is accomplished, a dwell time of 0.5 s is observed before moving to the next stage.
- Stage {5}: The stage holding the counterface is moved downwards to open the contact and to ensure the detachment between the mating surfaces.
- Stage {6}: The counterface plate returns to its starting point by executing a horizontal movement of the surface back to the initial position without load or contact with the arm, thus signifying the completion of one friction cycle.

All tests were conducted at room temperature (24 ± 1 °C) and a relative humidity of 37 ± 3%. Maintaining these environmental conditions throughout each test and repetition is crucial to ensure the accuracy and reproducibility of the results. Some of the tests were performed under dry conditions, while others were carried out in wet conditions with the addition of 1 mL of distilled water (DW). This allowed the examination of the effect of the presence of lubricant on the frictional behavior in the different configurations and allowed us to compare the results with those obtained under dry conditions.

## 3. Results and Discussion

As mentioned above, the PVS hexagon-patterned sample, as well as the flat reference sample, were both tested against three different counterfaces, i.e., (i) glass, (ii) gelatin, and (iii) chicken skin, under wet and dry conditions. The different test configurations considered in this study are shown in Table 2. All configurations were tested under a constant normal load of 5 N and three different sliding velocities, i.e., 5, 7.5, and 10 mm/s.

**Figure 7:**
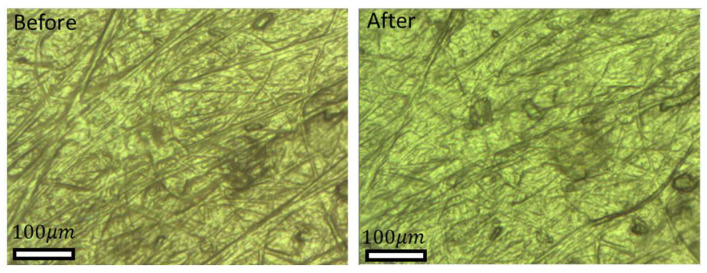
Optical microscope images for gelatin before and after friction test under dry conditions.

**Figure 8:**
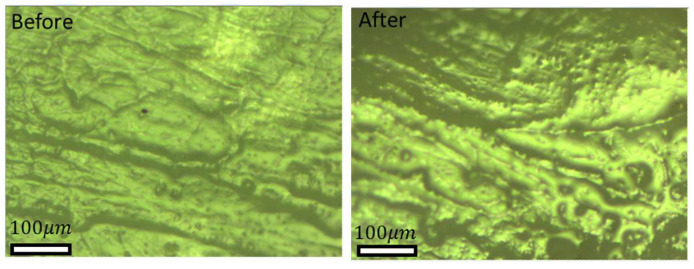
Optical microscope images for chicken skin before and after friction tests under dry conditions.

Figure 7 and Figure 8 show the friction track of the counterfaces, gelatin and skin, before and after friction under dry contact conditions for which the highest friction coefficients were obtained. It can be clearly observed that there was no significant wear trace. The same constatation of wear absence was noted on the textured and flat samples. This can be attributed to the specimens being made from Polyvinyl Siloxane (PVS), which has a high resistance to wear. Therefore, any wear that might occur is likely to happen in the soft counterfaces (gelatin and chicken skin), but it is expected that this would only occur after a very high number of friction cycles (much more than 10). Furthermore, the applied normal load was not very high (5 N), resulting in a relatively low contact pressure, and thereafter very limited, even inexistent, wear.

### 3.1. Analysis of a Single Friction Cycle

**Figure 9:**
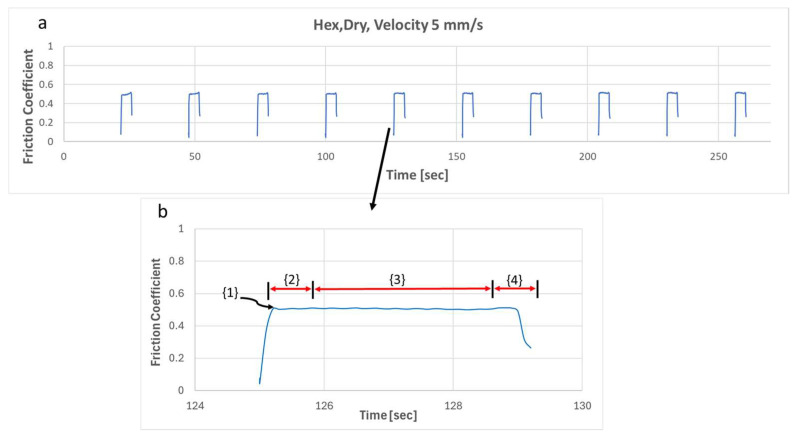
(**a**) Entire frictional behavior during 10 consecutive repetitions, and (**b**) a zoom-in on a single friction cycle with the following: {1} static coefficient of friction (µ_s_); {2} first 10% of dynamic friction; {3} 80% of the “stabilized” dynamic friction used to compute the average value of dynamic coefficient of friction (µ_d_); {4} last 10% of dynamic friction.

Each replication encompasses 13 consecutive repetitions, with calculations based solely on the data from the last 10 repetitions, excluding the initial three, which are considered as tests. Figure 9a features the results of 10 consecutive repetitions of test 4 obtained for the configuration of the hexagonal-patterned sample rubbed against glass counterface under dry conditions. The results demonstrate consistent repeatability across all 10 friction cycles. Figure 9b features the results of a single friction cycle (5th one) under the predefined operational and environmental conditions. As illustrated in Figure 9b, each individual repetition, both the static (µ_s_) and the dynamic (µ_d_) friction coefficients can be calculated. The static friction coefficient (µ_s_) is determined by dividing the measured friction force at the sliding inception (see {1} in Figure 9b) by the applied vertical force. On the other hand, the dynamic friction coefficient (µ_d_) is computed as the average friction force measured in the stabilized zone (middle of the sliding stock, see {3} in Figure 9b) divided by the applied vertical force.

### 3.2. Frictional Behavior of the Different Samples

**Figure 10:**
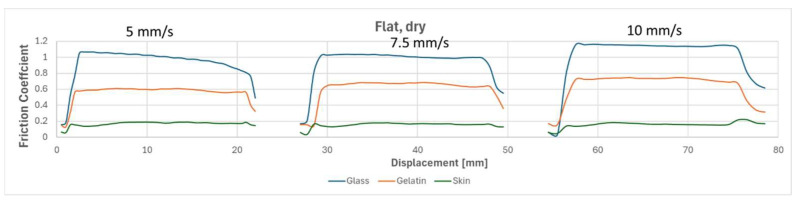
One friction cycle demonstrating the behavior of the flat reference at three speeds (5, 7.5, and 10 mm/s) against three different counterfaces under dry conditions.

**Figure 11:**
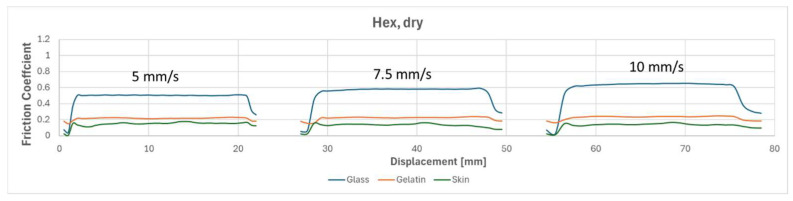
One friction cycle demonstrating the behavior of a hexagonally patterned specimen at three speeds (5, 7.5, and 10 mm/s) against three different counterfaces under dry conditions.

**Figure 12:**
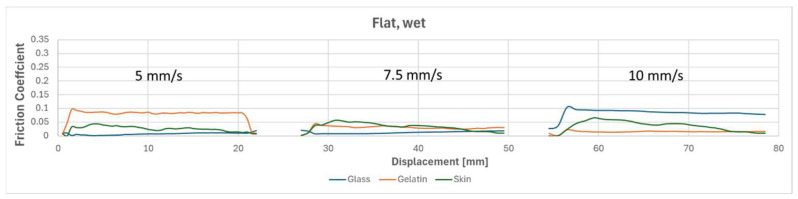
One friction cycle demonstrating the behavior of the flat reference at three speeds (5, 7.5, and 10 mm/s) against three different counterfaces under wet conditions.

**Figure 13:**
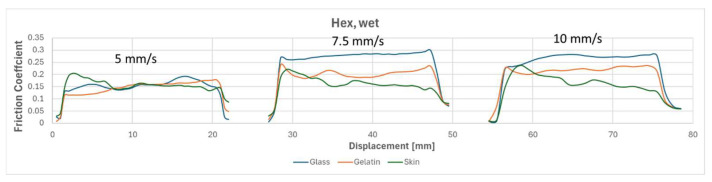
One friction cycle demonstrating the behavior of a hexagonally patterned specimen at three speeds (5, 7.5, and 10 mm/s) against three different counterfaces under wet conditions.

To compare the results of different tests, Figure 10, Figure 11, Figure 12 and Figure 13 overlay the frictional behavior during a single friction cycle (5th one) from each configuration, providing a visual comparison. The graphs highlight the distinct friction behavior of the flat reference and the micro-hexagonal-patterned PVS specimens when rubbed against the three different counterfaces, i.e., (i) glass, (ii) gelatin, and (iii) chicken skin, under dry and wet conditions. For instance, when comparing Figure 10 and Figure 11, it is evident that the friction coefficient of the flat specimen is generally greater than that of the hexagonally patterned specimen in dry conditions, regardless of the counterface and the sliding velocity. This can be explained by the greater real contact area between the flat sample and the counterfaces in comparison to the textured sample along with the absence of lubricant in the interface. However, under wet conditions with the presence of DW, a contrasting trend is observed. Figure 13, compared to Figure 12, shows a significantly higher friction coefficient for the patterned sample against all three counterfaces, regardless of sliding velocity. This phenomenon can be attributed to the fluid escaping into the micro-channels present between the hexagon subdivisions of the specimen due to the generated squeeze, thereby increasing the real contact area resulting in higher friction, as was suggested in references [32,40]. In contrast, for the flat specimen, the liquid is unable to escape and remains retained in the interface for a longer time, leading to extremely low friction coefficients due to a more developed hydrodynamic lubrication regime (see Figure 12). Furthermore, there was a significant fluctuation in the friction behavior against the chicken skin counterface, as observed in Figure 13. This can be attributed to the waviness features of the skin counterface, which induces a certain friction instability.

### 3.3. Discussion

**Figure 14:**
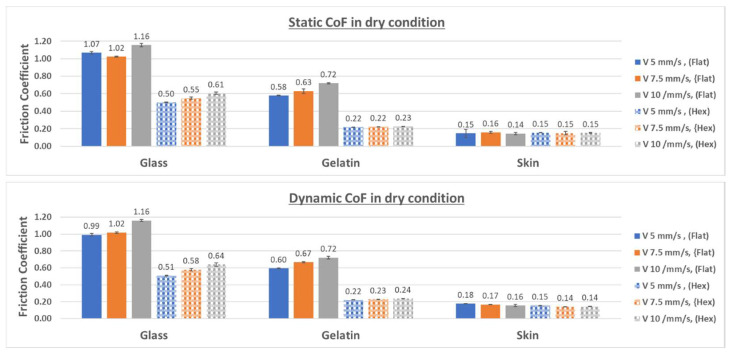
Static and dynamic CoF for all configurations under dry contact conditions.

To objectively compare the friction results from the different configurations, the average values of the static and dynamic coefficients of friction, as well as the relative standard deviations, were computed from the ten repetitions of each test configuration. Figure 14 presents the average values of the friction coefficients for the micro-hexagonal-patterned sample and the flat reference obtained against the three counterfaces under dry conditions.

As can be seen in Figure 14, both the static and dynamic coefficients of friction of the flat specimens tested against glass and gelatin in dry conditions are greater than those of their equivalent patterned specimens. The high friction in flat specimens can be explained by the greater real contact area in flat (untextured) samples, free of micro-channels, which is thought to increase the adhesive friction component. This difference in terms of friction between flat and textured samples is attenuated in the case of chicken skin, likely due to the rougher surface, thus diminishing the expected effect of the micro-channels.

In addition, comparing the results from all three material counterfaces, it can be seen that the coefficients of friction are highest on glass, followed by gelatin, and then chicken skin. This trend correlates well with the elastic modulus and yield shear stress value of the counterfaces, which is highest for glass, followed by gelatin and chicken skin. Since in dry contact conditions movement involves overcoming shear forces, materials with higher yield shear stress values have greater resistance to movement, resulting in higher friction coefficients. In addition, the significantly higher friction observed with glass compared to other configurations can be attributed to its smooth surface, which increases the real contact area and consequently enhances adhesive friction. When it comes to comparing the results of the soft counterfaces (gelatin and skin), the higher friction observed with the gelatin counterface in dry conditions can be related to its low surface roughness compared to that of the skin (see Figure 5), which is expected to generate greater surface contact and hence higher adhesive friction. The results show that variations in sliding velocities did not significantly impact the friction values observed under dry conditions for all three materials of the counterface, at least in the range of velocities considered in the present study. This finding tends to illustrate that, under these contact conditions, the samples have mostly elastic behavior fortified by the absence of lubricant in the interface.

**Figure 15:**
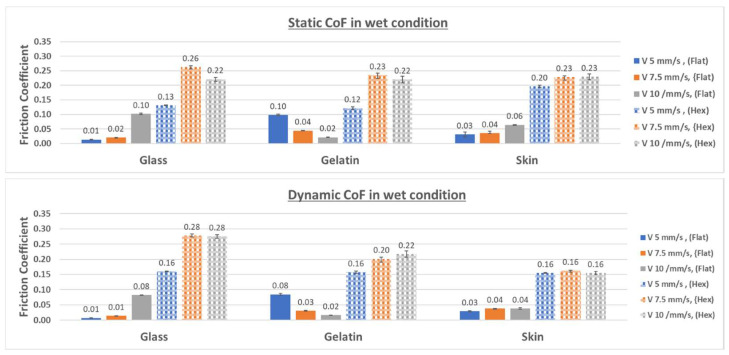
Static and dynamic CoF for all configurations under wet contact conditions.

Figure 15 presents the average values of the friction coefficients for the micro-hexagonal-patterned sample and the flat reference obtained against the three counterfaces under wet contact conditions.

Contrary to dry conditions, under wet contact with the presence of DW, the static and dynamic coefficients of friction for the patterned surface are much higher compared to those of the flat reference specimen across all counterfaces, as can be seen in Figure 15, albeit smaller when compared to those obtained with the patterned surfaces tested under dry conditions (compare with the results presented in Figure 14).

As can be seen in Figure 15, the static friction coefficient of the patterned surface tested against glass was up to 13 times greater than that of the flat specimen tested against glass. Similarly, against gelatin, the static friction coefficient of the patterned surface was higher by up to a factor of 11 compared to its flat reference specimen. When tested against chicken skin, the patterned surface exhibited a higher static friction coefficient by up to a factor of 6.6 compared to the flat reference specimen.

For the dynamic coefficient of friction, the patterned surface against glass demonstrated a maximum increase of a factor of 28 compared to the flat specimen against glass. Against gelatin, this coefficient was higher by up to a factor of 11, and against chicken skin, it was higher by up to a factor of 5.3.

The increased friction coefficients of the patterned surfaces compared to the flat ones under wet conditions can be attributed to different factors:

▪ Due to the generated squeeze pressure, the liquid can escape the interface between the mating surfaces into the micro-channels present between the hexagonal microstructures, therefore generating a greater contact area and thus a greater adhesive friction [32].
▪ The escape of the liquid from the interface reduces the lubrication effect, hence achieving friction-enhancing properties.
▪ In the presence of liquid in the interface, hexagonal microstructures can slightly deform locally under pressure, increasing the actual contact area between mating surfaces. This deformation enhances friction by creating additional contact points and intensifying the interaction between the surfaces.
▪ The hexagonal-patterned surface can trap liquid in its micro-channels, creating a thin film that adheres to the surface through capillary forces. This trapped water can increase the adhesive forces between the surfaces due to capillarity, thus enhancing friction [18].

Regarding the friction behavior observed against a gelatin counterface, in wet conditions, a contrast can be noticed. When tested against a flat specimen, there is a decrease in friction coefficient with increasing sliding velocity. On the other hand, when tested against the patterned specimen, the friction seems to increase with higher velocities. This opposite behavior suggests that the observed trends cannot be attributed solely to the viscoelastic properties of gelatin but are significantly influenced by the type of specimen (flat or patterned) used in the test. For the flat specimen, increasing the sliding velocity tends to capture more lubricant in the interface, and according to the Stribeck curve, since the normal load and lubricant viscosity are maintained constant throughout all the conducted tests, higher sliding velocities move towards mixed lubrication conditions, typically resulting in lower friction coefficients. For the patterned specimen, higher sliding velocities accelerated the movement of liquid into the micro-channels present between the hexagon subdivisions. This increased the real contact area and led to higher friction coefficients, as the fluid was squeezed into these channels more effectively.

Unlike the gelatin counterface, the variations in sliding velocity had no noticeable effect on the friction coefficient of the different chicken skin configurations in wet conditions. This is primarily due to the high roughness of the chicken skin surface, as is clearly visible in Figure 5. The high surface roughness implies that variations in velocity did not have a significant effect on the real contact area, and thus, it did not alter the friction coefficient from one test to another.

The results from this study suggest that the friction coefficients of the hexagonally patterned surface against soft counterfaces are comparable to or slightly lower than those against glass counterfaces. To our knowledge, this study and [40] are the first to investigate the friction behavior of hexagonal micropatterns against soft counterfaces. These promising findings shed light on the potential of hexagonal micropatterns and their possible integration into technologies where soft counterfaces are present, such as in medical devices, surgical instruments, and prosthetics that can benefit from increased friction in wet conditions for better control and stability.

## 4. Conclusions

The objective of this study was to analyze the frictional properties of biomimetic micro-hexagonal-textured surfaces interacting with soft counterfaces, specifically gelatin and chicken skin. To achieve this, friction tests were conducted using a customized biaxial tribometer at the Tribology and Microstructure Laboratory of Azrieli College of Engineering in Jerusalem.

The experiments involved preparing PVS specimens, both patterned and flat reference, and testing them against glass as a reference and against gelatin and chicken skin counterfaces under dry and wet conditions. These tests were performed at different sliding velocities.

Main findings:

1. Dry conditions:
  - The flat reference sample demonstrated higher friction coefficient values than those of the hexagonally patterned ones across all counterfaces.
  - A correlation was observed between the (1) yield shear strength and (2) roughness of the counterface tested and the friction coefficient, with glass exhibiting the highest friction coefficient, followed by gelatin and chicken skin.
2. Wet conditions:
  - The micro-hexagonal-patterned surface showed significantly higher friction coefficients compared to the flat reference sample.
  - The friction coefficient results against soft counterfaces were equal or slightly less in comparison to the glass counterface.
  - Increasing the sliding velocity had an indirect relationship with the friction coefficient values observed when a flat specimen was tested against a gelatin counterface. This can be explained by the tendency of the captured liquid between the interface to form a mixed lubrication regime, resulting in lower friction at higher velocities.
  - Increasing the sliding velocity had a direct relationship with the friction coefficient when the hexagonal-patterned specimen was tested against a gelatin counterface. This is due to the fluid escaping faster into the microchannels, increasing the real contact area and consequently increasing friction.
  - Variations in sliding velocity had no significant effect on the friction values against chicken skin due to its high surface roughness.

This study’s outcomes contribute valuable knowledge to the field of tribology, particularly in understanding the behavior of the hexagon micropattern structures and their interactions with soft counterfaces under wet contact conditions, which can be beneficial for future utilization in various applications where soft countersurfaces are present, such as in medical devices, surgical instruments, and prosthetics that can benefit from increased friction in wet conditions for better control and stability.

## Figures and Tables

**Table 1:** Roughness parameters values, *R_a_* and *R_t_*, of all three counterfaces used.

	Glass	Gelatin	Skin
*R_a_*	30 nm	0.9 µm	0.4 µm
*R_t_*	50–500 nm	84 µm	31 µm

**Table 2:** Test conducted with the respective configurations and conditions.

Test	Configuration		Contact Conditions
1	Flat reference sample (PVS)	Glass	Dry
2		Gelatin	
3		Chicken skin	
4	Hexagon-micropatterned sample (PVS)	Glass	Wet (DW)
5		Gelatin	
6		Chicken skin	

## Data Availability

Data are contained within the article.
